# The views of public service managers on the implementation of National Health Insurance in primary care: a case of Johannesburg Health District, Gauteng Province, Republic of South Africa

**DOI:** 10.1186/s12913-021-06990-4

**Published:** 2021-09-15

**Authors:** S D Murphy, S Moosa

**Affiliations:** Department of Family Medicine, Faculty of Health Sciences, University of the Witwatersrand, Johannesburg, South Africa

**Keywords:** Universal Health Coverage, National Health Insurance, Managerial capacity, Qualitative research, Decentralised governance, Primary care

## Abstract

**Background:**

The South African government is implementing National Health Insurance (NHI) as a monopsony health care financing mechanism to drive the country towards Universal Health Coverage (UHC). Strategic purchasing, with separation of funder, purchaser and provider, underpins this initiative. The NHI plans Contracting Units for Primary healthcare (PHC) Services (CUPS) to function as either independent sub-district purchasers or public providers and District Health Management Offices (DHMOs) to support and monitor these CUPS. This decentralised operational unit of PHC, the heartbeat of NHI, is critical to the success of NHI. The views of district-level managers, who are responsible for these units, are fundamental to this NHI implementation. This qualitative study aimed to explore district and sub-district managerial views on NHI and their role in its implementation.

**Methods:**

Purposive sampling was used to identify key respondents from a major urban district in Gauteng, South Africa, for participation in in-depth interviews. This study used framework analysis methodology within MaxQDA software.

**Results:**

Three main themes were identified: managerial engagement in NHI policy development (with two sub-themes), managerial views on NHI (with three sub-themes) and perceptions of current NHI implementation (with six sub-themes). The managers viewed NHI as a social and moral imperative but lacked clarity and insight into the NHI Bill as well as the associated implementation strategies. The majority of respondents had not had the opportunity to engage in NHI policy formulation. Managers cited several pitfalls in current organisational operations. The respondents felt that national and provincial governments continue to function in a detached and rigid top-down hierarchy. Managers highlighted the need for their inclusion in NHI policy formulation and training and development for them to oversee the implementation strategies.

**Conclusions:**

It appears that strategic purchasing is not being operationalised in PHC. NHI policy implementation appears to function in a rigid top-down hierarchy that excludes key stakeholders in the NHI implementation strategy. The findings of this study suggest an inadequate decentralisation of healthcare governance within the public sector necessary to attain UHC. District managers need to be engaged and capacitated to operationalise the planned decentralised purchasing-provision function of the DHS within the NHI Bill.

## Background

South Africa, one of the most unequal societies in the world, is an upper middle-income country that suffers a crippling quadruple burden of disease [1]. At the center of this crisis, South Africa’s fragmented healthcare system fails to cope with this disease burden, and further propagates societal inequity [2]. The extant bipartite health system was institutionalised through historical ethnic and socioeconomic deprivation and marginalization. The Apartheid regime established 14 separately-operating health departments [3] and passed legislature that favoured the privatisation of healthcare. These exclusionary structures have systematized the view of health as a commodity in the post-apartheid era and continue to drive excessive medical specialisation coupled with the bloom of myriad independent medical schemes. The dawn of democracy in 1994 heralded a social solidarity that sought to establish the basic human right to the progressive realisation of the access to equal and quality healthcare [4]. However, the prevailing apartheid legacy has continued to drive social inequity - South Africa spends nearly 9 % of its gross domestic product on healthcare [5], with more than 80 % of this allocation used to provide healthcare services to the top socioeconomic quintile [6]. The country’s fragmented healthcare system remains disease-oriented and perpetually disadvantages the poor with unequal access to care and catastrophic out-of-pocket expenditure.

The South African government is introducing a new healthcare financing and service delivery system - National Health Insurance (NHI) – to reorganize healthcare governance to achieve universal health coverage (UHC). UHC is advocated by the World Health Organisation [7] as a means for governments to improve health levels, reduce the burden of disease, cross-subsidise risk and enhance social equity through increased access to comprehensive services, increased population coverage, and financial protection. NHI intends to create a single financial pool for national risk-pooling and cross-subsidisation that will generate funds through an increased GDP expenditure on health, new payroll taxes, and increased income tax and value-added tax [4]. This fund looks to subsume the fragmented health insurance schemes and create a uniform, equitable social insurance scheme that standardises healthcare services across South Africa. UHC, through NHI, flips the view of health as a commodity to health as a basic human right, and represents a considerable reorganization of the healthcare service delivery model.

Although UHC is a laudable social construct, South Africa has failed several previous endeavours to implement structural and financial health reforms [2]. The NHI Bill acknowledges these failures to translate theoretical frameworks into actionable plans, and posits the devolution of healthcare governance from provincial health structures to district and subdistrict managers (DMs) as critical to successful NHI implementation [4]. The National Health Act of 2003 (NHA) [2] created the structures of the current public health system with national and provincial departments of health that sought to provide redress to the fragmented healthcare system. Within this legislative framework, the national health council oversees policy formulation and national priority setting, while provincial government is legislated (through the intergovernmental fiscal relation system) to receive the bulk of financing and is responsible for healthcare service delivery through the district health system as an agent of the province. Operational control of districts remains vested in provinces, with DMs appointed by provinces and local government facilities and staff being moved under provincial control. This top-heavy model has resulted in a detached governance structure that fails to meet both organisational standards of compliance [8] as well as the needs of South African citizens [2].

One of the central premises of the NHI Bill is that the devolution of governance to DMs will synergise top-down policy with the insight and leadership of DMs who face the complex realities of healthcare service delivery. To facilitate the reorganisation of the healthcare system, the NHI Bill repeals the previous legislative framework in the NHA and vests control of personal public services with DMs within their districts. Strategic devolution of governance, coupled with commensurate managerial capacitation, intends to empower DMs with the authority and expertise to implement gatekeeping (of entry and access to standardised healthcare services), establish clear referral pathways and reorient the service delivery towards comprehensive and integrative services through primary healthcare (PHC) re-engineering [4]. The Bill intends for the NHI fund to finance Contracting Units for Primary healthcare Services (CUPS) that will function either as sub-district purchasers, funded by the NHI fund, or as current sub-district public providers contracted with NHI to provide personal services. Contracting Units for PHC Services (CUPS) are the preferred organisational unit [9] (comprising an integrated network of district hospital, clinics or community health centres, and private providers) with which the NHI fund will directly contract to provide PHC services in a subdistrict. District Health Management Offices (DHMOs) will facilitate and coordinate PHC services and strategic purchasing [2] at the district level, directly accountable to the national department. Current DMs will function within DHMOs to support and monitor these CUPS [9, 10].

Longstanding UHC schemes such as the National Health Scheme (NHS) in the United Kingdom (often thought of as the archetypal UHC system) and the Medicare system in Canada have invariably improved national health and economic outcomes within their respective countries [11]. Stakeholders maintain that accountable and delegated management is the greatest predictor of successful UHC implementation [11] and both systems have reiterated the need for a ‘strategic vision’ at the level of district organizational structures [11], as well as the capacitation of local health managers (DMs) to improve their ability to enforce implementation. In the developing world, regions such as Asia, Latin America and sub-Saharan Africa have shown that inadequate decentralization of health system governance is crippling to UHC implementation strategies [7, 12, 13]. In contrast, UHC successes in countries such as Thailand and Indonesia, nations with low gross national income per capita, have been attributed to sustained federal investment in devolving governance to the district structures of healthcare [14]. Similarly, the Cuban health care system, which relies predominantly on CUPS, has shown that the decentralised approach to healthcare allows targeted strategies that address community-specific needs, increase the equity of care, and promote managerial flexibility and accountability [12].

In the South African context, the first phase of NHI (2012–2017) piloted health services strengthening initiatives targeted at PHC – the “heartbeat of NHI” [15]. Ten interventions were implemented across eleven pilot sites. A recent external evaluation of phase 1 was released in 2019 [2] which showed mixed results across the sites. The authors noted several shortfalls in critical governance components, similar to those in several countries already implementing UHC [7, 12, 16], due to an inadequate decentralision of governance. The findings of this review support previous studies showing that a recurrent obstacle to NHI implementation is its dependence on the detached and centralized governance model [17]. A recent report by the Office of Health Standards Compliance [8] (OHSC) showed that primary healthcare clinics yielded a 47 % compliance across seven domains of quality. The domain of leadership and governance scored 47 % - classified as ‘non-compliant’ and ‘requiring urgent intervention’. Another prominent concern is that the lack of transparency and accountability, inherent in a centralized model of governance, allows corruption (in the form of irregular expenditure) to remain unchecked - the OHSC audited nearly 45 % of health care expenditure as irregular [8].

It is evident that the effective devolution of governance will play a vital role in the mechanisms intended to facilitate the implementation of NHI. DMs need to be fully aware of, and engaged in, the NHI structures, processes and rollout plan. Despite the growing appreciation that the success of UHC interventions is critically dependent on decentralized management, a thorough literature review yielded no studies evaluating this. We aimed to explore the views of DMs on NHI, as well as their engagement in policy development and implementationvand health system reorganisation as mandated by the NHI Bill.

## Methods

This was an exploratory qualitative study with a focus on applied policy. Our study was conducted within the Johannesburg Health District, one of Gauteng province’s seven health districts, between 2020 and 2021. This district was selected as it is a major district with geopolitical proximity to provincial and national governance structures. Delays and challenges in obtaining ethics approval from several district ethics committees hindered a planned evaluation of other Gauteng districts.

 A list of DM personnel was obtained from the district ethics committee to facilitate purposive sampling of data rich participants. Sampling was conducted through a framework of stratified random sampling so that a balanced mix of DMs from all backgrounds would be sampled. Ethics committee approval of the study was obtained from the University of Witwatersrand’s Human Research Ethics Committee (M191046), as well as the Research Committee of the Johannesburg Health District (GP_202006_048).

In-depth interviews were selected as the method of data collection to allow respondents to develop their own description of their perspectives and experiences. The principal investigator (SAM) conducted all interviews. Both researchers had undergone several courses on qualitative interviewing skills through their university and conducted previous qualitative work. Further, the senior researcher has previously published several qualitative studies [18, 19]. Email invitations which included a participant information sheet were sent to prospective participants. Due to the COVID-19 pandemic, interviews were conducted with consenting participants by telecommunication software as regulated by the ethics committee. The four core questions used in the interviews are described in Table 1. Interviews were recorded via a dictaphone and transcribed verbatim into electronic text format. The concepts of ‘information power’ and ‘informational redundancy’ [20] informed pragmatic sampling saturation. Saturation was reached following seven in-depth interviews, although a total of ten interviews were conducted. Transcripts were sent back to participants for member-checking. No changes were made following member checks. Transcripts were then imported into computer-assisted qualitative data analysis software (MaxQDA [21]) to facilitate robust and explicit data analysis. Data analysis followed the five-step approach of Framework Analysis [22]: familiarization, framework formulation, coding and indexing, charting, and mapping and interpretation. Peer-checking of the thematic analysis, as well as the greater framework analysis, was performed by the second researcher. The researchers met regularly to cross-examine analyses. No divergent interpretations arose during this process. Measure to ensure the trustworthiness of this work are listed under Table 2 below.

**Table 1:** Overarching questions in the interview schedule

Nº	Question
1.	What are your views on National Health Insurance?
2.	Can you tell me about your engagement in NHI policy development?
3.	What is your perception of the implementation of NHI?
4.	Is there any other view or thought you would like to express?

**Table 2:** Measures to ensure study trustworthiness

Component of Trustworthiness	Measures
Credibility	This study will used validated Framework Analysis method. Critical reflexivity was used during interviews with thick descriptions of data to facilitate internal validity.
Transferability	Detail of the research setting with an audit trail is provided for future research and cross comparison.
Confirmability	Computer assisted qualitative data analysis software was used to leave a paper trail alongside reflexive commentary. Quotes are used for in vivo description.
Dependability	Study design and methods are made explicit so that researchers can emulate the study design.

## Results

Ten district managers were interviewed for the study. Participant ages and work experience within healthcare services ranged between 35 and 65 years and 6–42 years respectively. However, the duration of their current position as a manager ranged from 1 month to 6 years. Six participants were female and four were male. Seven participants were DMs, and three participants were sub-DMs. All the research participants were employed within the Johannesburg Health District in Gauteng. The respondent profiles are summarised in Table 3 below.

**Table 3:** Respondent Profiles

Identifier	Ethnicity	Gender	Designation	Duration in Current Position
Respondent 1 (R1)	African	Male	DM	1 month
Respondent 2 (R2)	African	Male	DM	3 months
Respondent 3 (R3)	Coloured	Female	DM	6 months
Respondent 4 (R4)	White	Female	DM	> 1 year
Respondent 5 (R5)	African	Female	sub-DM	> 1 year
Respondent 6 (R6)	Indian	Male	DM	> 1 year
Respondent 7 (R7)	African	Male	sub-DM	> 1 year
Respondent 8 (R8)	African	Female	DM	4 months
Respondent 9 (R9)	African	Female	sub-DM	> 1 year
Respondent 10 (R10)	Coloured	Female	DM	3 months

**Fig. 1:**
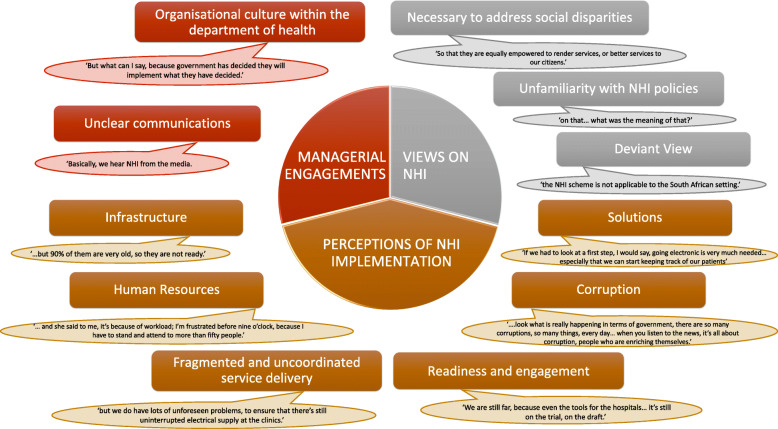
Summary of findings

Three main themes were identified, represented in the figure below: managerial engagement in NHI policy development (with two sub themes), managerial views on NHI (with three sub-themes), and perceptions of current NHI implementation (with six sub-themes) (Fig. 1).

### Managerial engagement in policy development

#### Communications received and engagement with NHI rollout

The respondents described ambiguous experiences regarding their engagement in policy development. Respondents cited uncertainties around roll-out plans as well as their own managerial responsibilities that they attributed to a near absence of communication around NHI from central governance structures. Several respondents were aware of other policy implementations such as PHC re-engineering and Ideal Clinics [4] as part of the greater NHI communication and implementation from national and provincial government, but struggled to see the bigger picture, often leaving them with more questions than answers:

The third respondent (R3) stated, ‘You don’t get the total information, you get an introduction of what you might do for their target.’

The seventh respondent (R7) declared, ‘[We are] at the coalface of service delivery… to engage and to request for our inputs. I think it was not done properly.’

The fifth respondent (R5) said, ‘Basically, we hear NHI from the media; we haven’t really had people come in and talk to the people.’

#### Organisational culture within the department of health

Some respondents depicted their absence of engagement and the one-sided narrative around NHI as a recurrent organisational practice. Other respondents felt disillusioned by the lack of senior managerial engagement and commitment to NHI:

The seventh respondent (R7) expressed, ‘But what can I say, because the government has decided they will implement what they have decided to do.’

The tenth respondent (R10) declared, ‘But really, I would not know why the department is not giving it the attention that it needs.’

### Managerial views on NHI

#### Managerial views on NHI policies and documents

A prevailing theme across all of the interviews was that respondents were unfamiliar with NHI documents, policy and law. This finding supports the overarching theme of poor managerial engagement described above.

The second respondent (R2) asked, ‘on that… what was the meaning of that?’

The fourth respondent (R4) said, ‘I don’t know, I really don’t have an idea…’.

The interviewer frequently needed to describe the content and concepts contained within the NHI policy document to the managers. Consequently, interviewees often lacked the insight to provide meaningful commentary.

The fifth respondent (R5) said, ‘Maybe, I’m not so sure… So I don’t know, I don’t want to comment on something that I’m not so sure of… how is it going to work, or what.’

The sixth respondent (R6) stated, ‘I’m not sure by facility, or … by municipality, or the service provider… then to, I suppose…So I’m not too sure, I haven’t looked at the detail.’

#### The need to address social disparities

Despite a lack of familiarity with NHI policies, most managers viewed NHI as a mandatory social intervention to redress social inequities and unequal access to quality healthcare. This was coupled with the impression that NHI would redistribute resources from the private health facilities to serve as a panacea to current public sector challenges:

The first respondent (R1) maintained, ‘The issue of overcrowding on public institutions, I think it will also reduce that… which will actually reduce on the number of litigations.’

The first respondent (R1) added, ‘So that they are equally empowered to render services, or better services to our citizens.’

The fourth respondent (R4) declared, ‘I think NHI aims to ensure that the issue of social solidarity between the healthy and the sick, those who have and those who have not.’

#### Views on the current healthcare system

There was a singular deviant case that saw the NHI as a scheme that was not applicable to the South African setting. The participant regarded the current private health system as adequately suited to the South African context and felt that more meaningful interventions could be made towards improving existing public health services.

### Perceptions of NHI implementation

#### Concerns around the current healthcare infrastructure and policies

All participants regarded the current public healthcare system as not ready to implement NHI. Respondents expressed concerns around infrastructure that had aged and not been maintained,

The first respondent (R1) maintained, ‘You cannot be sitting with a hospital that is built 50 years back… but 90 % of them are very old, so they are not ready.’

The sixth respondent (R6) declared, ‘The second thing is, remember, our health information system, our epidemiological data, is limited; how do they begin to develop appropriate plans.’

Furthermore, respondents emphasized that the health system was not standardized and access to quality care varied across the country:

The fifth respondent (R5) asked, ‘What about the areas where people still need to travel about a hundred kilometres to access a clinic?’

Respondents expressed several concerns around current performance and quality appraisal mechanisms. They saw current indicators as invalid and unreliable - often misrepresented to appease executives:

The second respondent (R2) maintained, ‘There will be no questions to say, how did you reach this target, how did you do whatever. As long as you are presenting, to say, I’m at 55 %, when the target was 50 %.’

#### Views on readiness to implement NHI

Most respondents felt disempowered and lacked clarity on their role in the NHI. They expressed concerns around poor quality and one-way communication, as well as not knowing what the requirements or procedures would be. Further, respondents described doubt around the readiness of the health system to implement the NHI policies due to experiences with several long-standing policies and laws that had not yet been effectuated:

The fifth respondent (R5) said, ‘We are still far, because even the tools for the hospitals… it’s still on the trial, on the draft.’

#### Concerns around corruption within the governance structures

A major theme that emerged was that of corruption throughout all tiers of governmental structures. Distrust towards government seemed to be magnified when managers discussed the proposed increased fiscal expenditure on health in the NHI Bill:

The third respondent (R3) affirmed, ‘Well, I definitely don’t think that the current structure should govern it.’

The fifth respondent (R5) declared, ‘When you look what is really happening in terms of government, there are so many corruptions, so many things, every day… when you listen to the news, it’s all about corruption, people who are enriching themselves.’

Respondents suggested a reform of managerial structures that fostered good governance, transparency and accountability:

The ninth respondent (R9) said, ‘… but if ever you don’t have a consequence management approach, and accountability, things can just fall through, without anybody really paying attention or taking responsibility.’

#### Human resource challenges

Respondents held that challenges around good governance were exacerbated by deficits in human resource management at national and provincial levels. Respondents expressed concerns around staff retention and ineffective devolution of management. Further, respondents described an absence of consensus orientation or succession planning towards NHI implementation:

The first respondent (R1) said, ‘What we’re supposed to be doing now, is to do succession planning and align it to National Health Insurance.’

Several respondents highlighted challenges around understaffing within the district, resulting in overworked and demotivated employees:

The first respondent (R1) declared, ‘… and she said to me, it’s because of workload; I’m frustrated before nine o’clock, because I have to stand and attend to more than fifty people… then I ask in private, in private I’ll be allocated only five…’.

#### Fragmentation and incoordination of services

Several respondents expressed that healthcare service delivery systems functioned in a siloed fashion. Respondents felt that patient care was poorly coordinated between levels of care and referral pathways were poorly established. Further, beyond thehealth system, managers described the need for them to redirect energy and resources to ancillary supportive services to compensate for sub-optimal service delivery from other national and provincial departmental counterparts:

The fourth respondent (R4) stated, ‘So we do have the generators now, but we do have lots of unforeseen problems, to ensure that there’s still uninterrupted electrical supply at the clinics.’

The sixth respondent (R6) said, ‘But apart from making sure that the service standards are complied to… we need to make sure that other systems are in place… adequate for the work we have to do for that particular community.’

#### Potential avenues to facilitate NHI implementation

Participants emphasized the need for national-level government to facilitate the digitalisation of healthcare to map community profiles which would allow for tailored health service planning as well provide an avenue of communication to all stakeholders. Respondents also spoke to the development of a booking system to optimize the flow of patients throughout facilities and improve user experience:

The third respondent (R3) maintained, ‘If we had to look at a first step, I would say, going electronic is very much needed… especially that we can start keeping track of our patients’.

The eighth respondent (R8) said, ‘But if proper SMSs or appointments were sent… reduced waiting time will definitely improve services.’

Respondents held that national government needed to expand multilateral collaborative efforts alongside in-house managerial capacitation to nurture comprehensive and inclusive NHI implementation strategies that reduced duplication and fragmentation of health services:

The first respondent (R1) declared, ‘… trained, specifically on National Health Insurance; and not from one discipline, it must be a multi-tasked or disciplinary team.’

The fourth respondent (R4) maintained, ‘So I think it’s to listen to the community, hear what all the stakeholders have to say… to what could be better solutions.’

Lastly, managers felt that national government should iteratively appraise NHI strategies through shared international learning and reflection to guide efficient local NHI implementation:

The sixth respondent (R6) declared, ‘I think we’re very fortunate in the sense that we can learn from lessons learnt in other countries.’

## Discussion

In this study we have described the evidence-based need for the Ministry of Health to raise the awareness, readiness and engagement of DMs to operationalize the pillars of UHC - effective health systems management is determined by a strategic managerial group identity across the levels of health service management. The NHI, as a radical reformation of the South African health sector, requires unison of vision, participation and consensus orientation, transparency and accountability. The NHI bill [4] states that the DHS is responsible for forming and collaborating with implementation structures such as DHMOs and CUPS. Capacitated DHMOs and CUPS are the bedrock of strategic purchasing that aims to address health system fragmentation, improve healthcare infrastructure, as well as enable accountable and transparent financial management.

The results of this study show that managerial engagement in NHI policy development and implementation remains [17] inadequate. Managers failed to appreciate the implications of the NHI Bill at the operational unit of PHC – the DHS – and lacked insight into the decentralised nature of contracting and financial management in strategic purchasing, the use of capitation, or that private providers may be in the expected mix of personal services provided. The deficits in managerial awareness and capacity, coupled with their exclusion from engagement of policy formulation and rollout, is contradictory to the apparent purpose of the NHI Bill to empower the DHS to assume responsibility for the needs of predefined municipal areas. Joint learning from Asian [14], Latin American [12] and African [13, 23, 24] regions has shown that incomplete operationalization of the DHS hinders UHC implementation strategies [10] and leads to wasteful expenditure. Conversely, success stories from countries such as Cuba [12], Thailand and Indonesia [7] show that UHC can be successfully implemented in the face of fiscal austerity, when sustained investment in decentralised governance strategies that enabled CUPs and strategic purchasing. Similarly, several African countries such as Uganda and Ghana have effectively used decentralised governance strategies to facilitate UHC success [13].

Notwithstanding the overall lack of familiarity with NHI policies, most managers viewed NHI as a mandatory social intervention to redress social inequities. However, there was an underlying notion amongst the respondents that NHI is a panacea to current public sector challenges by simple redistribution of resources from the private sector. This paradigm fails to appreciate the strategic and collaborative governance needed to address health system fragmentation and has resulted in corruption, wasteful expenditure and ultimately UHC failure in countries such as Kenya and Nigeria [9, 25].

Despite the abovementioned concerns, select studies have demonstrated that NHI intervention pilots have the potential to markedly improve health system performance [2] within resource constraints. These achievements were most evident in districts where governance was effectively assigned to the respective DHS. It is clear that when the policies within the NHI Bill are effectively applied, definitive progress can be made towards the attainment of UHC. Sustained federal investment in the DHS is necessary to operationalise NHI policies and provide redress to the fragmented and inequitable healthcare system.

### Study limitations

Only one district (of seven) in Gauteng was sampled for this study - a large urban district that is well-capacitated and in close contact with national and provincial governing bodies. The findings of this study do not accurately reflect all districts of South Africa, especially rural areas.

### Recommendations

This study should be repeated across the spectrum of rural and urban districts to corroborate findings and expand on contextual nuances for community-specific health system planning. Similarly, the qualitative findings of this paper could inform the constructs (by means of principal component analysis) for future quantitative questionnaires which, once validated, could be used for widespread evaluation of managerial decentralisation. Further, this could be coupled with explanatory models to guide strategic interventions targeted at healthcare governance.

Recent studies have illustrated the efficacy of brief training interventions that empower DHS managers to effectively govern healthcare services [2, 10]. It is recommended that future strategies for NHI implementation include specific engagement and capacitation of the managers of all 52 districts within South Africa to prepare for contracting at that level. In the current (second) phase of NHI implementation, National policy should employ validated frameworks [10, 16, 17] of health system governance and utilise existing bodies, such as the OHSC, to establish and enforce accurate indices of decentralised governance and health within each DHMO and their respective CUPS. Managerial capacitation could be targeted through human resource for health interventions [7, 14, 26] such as managerial fellowships, mentoring, internships and succession-planning. Directly engaging DMs will coordinate simple top-down assumptions with bottom-up insights (into the complex realities of service delivery) to focus and synergise available resources towards improving health outcomes.

## Data Availability

The dataset of this work is available in the Zenodo repository, 10.5281/zenodo.4765125, and can be accessed on reasonable request to the corresponding author.
